# The effect of high frequency steep pulsed electric fields on in vitro and in vivo antitumor efficiency of ovarian cancer cell line skov3 and potential use in electrochemotherapy

**DOI:** 10.1186/1756-9966-28-53

**Published:** 2009-04-22

**Authors:** Xiao-Jun Yang, Jun Li, Cai-Xin Sun, Fei-Yun Zheng, Li-Na Hu

**Affiliations:** 1 Department of Obstetrics and Gynecology, The First Affiliated Hospital of Wenzhou Medical College, Wenzhou, Zhejiang province 325000, PR China; 2 Department of Obstetrics and Gynecology, The Second Clinical Medical Institute of North Sichuan Medical College, Nanchong, Sichuan province 637000, PR China; 3 Key Laboratory of High Voltage Engineering and Electrical New Technology, Ministry of Education, Electrical Engineering College of Chongqing University, Chongqing 400044, PR China; 4 Department of Obstetrics and Gynecology, The Second Affiliated Hospital, Sichuan University, Chengdu, Sichuan province 610000, PR China

## Abstract

**Background:**

Patients received electrochemotherapy often associated with unpleasant sensations mainly result from low-frequency electric pulse induced muscle contractions. Increasing the repetition frequency of electric pulse can reduce unpleasant sensations. However, due to the specificity of SPEF, frequency related antitumor efficiency need to be further clarified. The aim of this study was to compare in vitro cytotoxic and in vivo antitumor effect on ovarian cancer cell line SKOV3 by SPEF with different repetition frequencies. Explore potential benefits of using high frequency SPEF in order to be exploitable in electrochemotherapy.

**Methods:**

For in vitro experiment, SKOV3 cell suspensions were exposed to SPEF with gradient increased frequencies (1, 60, 1 000, 5 000 Hz) and electric field intensity (50, 100, 150, 200, 250, 300, 350, 400 V/cm) respectively. For in vivo test, SKOV3 subcutaneous implanted tumor in BALB/c nude mice (nu/nu) were exposure to SPEF with gradient increased frequencies (1, 60, 1 000, 5 000 Hz) and fixed electric field intensity (250 V/cm) (7 mice for each frequency and 7 for control). Antitumor efficiency was performed by in vitro cytotoxic assay and in vivo tumor growth inhibition rate, supplemented by histological and TEM observations. Data were analyzed using one-way ANOVA followed by the comparisons of multiple groups.

**Results:**

SPEF with a given frequency and appropriate electric field intensity could achieve similar cytotoxicity until reached a plateau of maximum cytotoxicity (approx. 100%). SPEF with different frequencies had significant antitumor efficiency in comparison to the control group (P < 0.05). However, there was no difference in tumor responses among test groups (P > 0.05). Histological and TEM observations demonstrated obvious cell damages in response to SPEF exposure. Furthermore, SPEF with 5 kHz could induce apoptosis under TEM observations both in vitro and in vivo.

**Conclusion:**

SPEF with high frequency could also achieve similar antitumor efficiency which can be used to reduce unpleasant sensations in tumor electrical treatment. Our research proposed potential applications of using high frequency SPEF in clinical cancer treatment.

## Background

Electric field is a new biomedical engineering technique which can be used as electrochemotherapy, tumor ablation, or intracellular electromanipulation respectively [1,2]. The biological basis of electrohemotherapy is the combination of reversible membrane electroporation caused by weak intensity microsecond electric pulse and subsequent enhanced intracellular drug-uptake such as bleomycin and cisplatin for their cytotoxicity [3]. Alternatively, distinct from electrochemotherapy, irreversible membrane electroporation induced by intensive energy microsecond electric pulse can be used alone to implement tumor ablation directly without any cytotoxic drugs [4-6]. Furthermore, different from microsecond electric pulse, nanosecond electric pulse decreases its effect on plasma membrane and imposes electric force on multiple subcellular structures known as intracellular electromanipulation, which can be used in cancer treatment, gene therapy and wound healing [7]. Therefore, electric field possesses parameters related different biophysical effects. However, to the best of our knowledge, few researchers have involved any information about the biophysical effects regarding the combined application of microsecond and nanosecond duration electric pulse in cancer treatment.

Our group has dedicated to investigate antitumor effects of SPEF for many years. The distinct characteristic of this exponential decayed SPEF was that the rising period was shortened to the nanosecond level which contains abundant high frequency electromagnetic components (we call it steep pulsed electric fields), and the descending period remained in the microsecond level which contains lots of low frequency electromagnetic components [8]. Therefore, this specially designed SPEF composed of a dual component type of pulse, which different from microsecond duration, low repetition frequency electric fields typically used in electrochemotherapy. For the first time, we confirmed that the combined effect of micro- and nano-second electric pulse contained in SPEF could destroy cancer cells effectively through reversible or irreversible membrane electroporation [8-12] or trigger various biophysical responses within caner cells [13]. Furthermore, the killing effect of SPEF depended on pulse parameters, excessive electric field intensity could cause extra damage to surrounding normal tissue [14].

Repetition frequency of electric pulses was considered to have a close relationship to the contraction of muscle, which can lead to painful burning sensation in electrochemotherapy and often complained by many patients [15]. Increasing the repetition frequency of electric pulse delivery can reduce unpleasant sensations that occur in electrochemotherapy [15]. On the other hand, with respect to pulse frequency on antitumor efficiency, authors report that microsecond duration electric pulse with high repetition frequency actually doesn't decrease its antitumor efficiency in electrochemotherapy [16,17]. However, besides the pulse frequency that induces unpleasant sensations during electrochemotherapy, pain sensation also depends on pulse parameters such as pulse amplitude, number, duration, and shape of the pulses [18]. Therefore, due to the specificity of SPEF, further studies were still necessary to elucidate the effects of frequency related antitumor efficiency by the dual component type of pulse in SPEF.

In this study, we primarily aimed to compare in vitro cytotoxic and in vivo antitumor effect on ovarian cancer cell line SKOV3 by SPEF with different repetition frequencies. Our objective was to explore the effect of such electric pulses in order to be exploitable in electrochemotherapy. We reported in the article that SPEF with high repetition frequency (5 kHz) can also achieve similar levels of in vitro and in vivo antitumor efficiency. Furthermore, SPEF with 5 kHz could induce apoptosis under ultrastructural observations both in vitro and in vivo. It is hoped that this study would be helpful to evaluate the potential use of high frequency SPEF to reduce unpleasant sensations without decreasing therapeutic effect in clinical tumor electrical treatment. The conclusions can finally lead to new therapeutic approach in electrochemotherapy.

## Materials and methods

### Materials

#### Cell Culture

Human ovarian cancer cell line SKOV3 (Shanghai Biochemical Institution, Shanghai, China) was initially cultured in RPMI-1640 medium supplemented with 2 mM glutamine, 10% fetal bovine serum (FBS), 2% penicillin and streptomycin, and were maintained at 37°C and 5% CO_2_. Fetal bovine serum, RPMI-1640, MTT, DMSO, were provided by Sigma Company (Sigma-Aldrich, Inc St. Louis, MO, USA). Na-phenobarbital was provided by Fuyang Pharmaceutical Factory (Anhui, China).

#### Tumor Formation in BALB/c nude mice

BALB/c nude mice (nu/nu) (n = 35, 8-week-old, weighing: 25–28 g) were used for this study. Mice were kept at constant room temperature (25°C) with a natural day/night light cycle under SPF conditions with food and water provided ad libitum. Before experiments, all rats were subjected to an adaptation period of at least 10 days, without fungal or other infectious disease at the beginning of experiment. Animals were maintained in accordance with the principles outlined in the National Institute of Health Guide for the care and use of laboratory animals. Mice were provided by the Medical Experimental Animal Administrative Committee of Wenzhou Medical College, China (animal certification number: SCXK-20020001).

Solid subcutaneous tumor, located right rear limb in mice, was initiated by an injection of 2 × 10^6 ^SKOV3 cells in 0.2 ml 0.9% NaCl solution. The viability of the cells was over 95% as determined by a trypan blue dye exclusion test. Then tumor tissue was cut and implanted subcutaneously to establish tumor bearing mice. Six to 10 days after implantation when subcutaneous tumor nodules reached approximately (120.5 ± 18.2) mm^3^, tumor model was successfully established and subjected to electric fields stimulation protocols.

#### SPEF Exposure System

SPEF generator was designed by Sun et al., in the key laboratory of high voltage engineering and electrical new technology of Chongqing University [9]. The pulse curve was in form of unipolar exponential decay with the utmost voltage peak value 1000 V, pulse rise time ranging from 90–180 ns, pulse total duration 1–20 μs, and the frequency 1 Hz–5 kHz. Parameters in combination produced desired energy-controllable SPEF.

#### Electric Fields Stimulation Protocols

**Figure 1:**
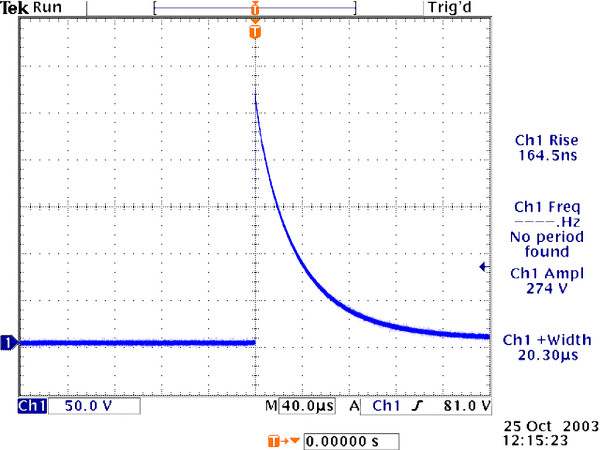
**Typical waveform of SPEF captured by Tektronix TDS3032B Oscilloscope**.

We used Tektronix TDS3032B Oscilloscope to monitor SPEF output and typical waveform captured referred to Figure 1. The parameters used for in vitro experiment referred to Table 1: eight unipolar exponential decay pulses with each 20 μs duration (rise time was 160 ns), with amplitudes from 50 to 400 V/cm, and pulse repetition frequencies of 1, 60, 1 000, 5 000 Hz were delivered (cell exposure time was 30 minutes).

**Table 1:** The parameters of SPEF used in SKOV3 cell suspensions.

Test group	Frequency (Hz)	Intensity (V/cm)	Rise time (ns)	Duration (μs)	Stimulation time (minutes)
Group 1	1	50, 100, 150, 200, 250, 300, 350, 400	160	20	30
Group 2	60	50, 100, 150, 200, 250, 300, 350, 400	160	20	30
Group 3	1 000	50, 100, 150, 200, 250, 300, 350, 400	160	20	30
Group 4	5 000	50, 100, 150, 200, 250, 300, 350, 400	160	20	30

In the first procedure, each intensity constituted a separate experiment contained in a certain test group, and cell exposure time was 30 minutes for each intensity corresponding to a given frequency.

The parameters used in SKOV3 implanted tumor referred to Table 2: eight unipolar exponential decay pulses with each 20 μs duration (rise time was 160 ns), with electric field intensity 250 V/cm, and pulse repetition frequencies of 1, 60, 1 000, 5 000 Hz were delivered (cell exposure time was 30 minutes).

**Table 2:** The parameters of SPEF used in SKOV3 implanted tumor.

Test Group	Frequency (Hz)	Intensity (V/cm)	Rise time (ns)	Duration (μs)	Exposure time (minutes)
test 1	1	250	160	20	30
test 2	60	250	160	20	30
test 3	1 000	250	160	20	30
test 4	5 000	250	160	20	30

In the second procedure, each frequency constituted a separate experiment, and tumor exposure time was 30 minutes for each frequency.

In this paper, we adjusted, the frequency of the pulses by changing the interval between two consecutive pulses in a train, and then keeping both the duration and number of pulses constant. Cell culture and SPEF-exposure were performed at room temperature (25°C) and data analysis was conducted in a blinded fashion.

### Determination of Frequency Related Antitumor Efficiency

#### In Vitro Cell Exposure and Cytotoxicity of SPEF

SKOV3 cells were digested with 0.25% trypsin and resuspended into steriled 24-well culture plate with average cell density being 1 × 10^5 ^cells/well. This self – made 24 – well culture plate was equipped with an array of platinum needle electrodes (0.3 mm in diameter, 10 mm long, 10-mm apart) mounted on a plastic holder to keep the distance constant and reproducible, with each well correspond to a pair of parallel electrodes connections to SPEF generator. This device can perform repeated experiments to ensure consistency and repeatability of the testing results. Control cells in 24-well plates received no electric stimulation.

After each exposure to a combined frequencies and electric field intensity (Table 1), for each test group, cytotoxicity of SPEF on SKOV3 was evaluated by MTT assay. Cells were then incubated with MTT (5 mg/ml) for 4 hours and DMSO (0.1%, V/V) for 10 minutes to perform MTT assay [19]. Optical density (OD) was determined at 490 nm by using a microplate reader (BIO-RAD, model 550, USA). Non-treated cells in self – made 24 – well culture plate served as control, and also got MTT assay in the same way. For each test group, a corresponding cytotoxicity was calculated and the data shown were representative of the mean of at least three independent experiments on different days.

Cytotoxicity was determined according to the equation: % cytotoxicity = (control value - experimental group)/(control value) × 100%. Moreover, drew the curve of cytotoxicity of SPEF for SKOV3 under different frequencies and electric field intensity.

#### In Vivo Tumor Exposure and Tumor Volume Inhibition Efficiency

Twenty-eight established tumor bearing mice were randomly divided into four experimental groups (7-mice in each frequency group) and subjected to a relevant SPEF exposure protocol using platinum needle electrodes (0.3 mm in diameter, 10 mm long, 10-mm apart) on day 0 (Table 2). Another 7-mice in non-exposure group served as control. All mice received anaesthetized by Na-phenobarbital (i.p.:30 mg/kg body weight) during SPEF exposure. Then mice were maintained under SPF conditions.

Tumor size was measured in all mice accurately with a digital calliper before and every day after SPEF exposure. Tumor volumes were calculated from the equation: V = π·A·B·C/6 (mm^3^) (A length, B width, C height) [17]. On the 26^th ^day after SPEF exposure, tumor volume inhibition rate was calculated by using the equation: Inhibition rate = (1- tumor volume of test group/tumor volume of control group) × 100% [20]. Moreover, drew the tumor growth curve of tumor volume according to observation time for each frequency group.

#### Light and TEM Observation

After in vitro exposure to SPEF, SKOV3 cell samples in each experimental group were subjected to multi-step processes and finally rendered to TEM observation (H-600, HITACHI, Hitachi Ltd, Japan). Non-treated control cells also get TEM assay in the same way.

After in vivo exposure to SPEF, one mouse from each experimental group and control group were fed for 3 days before received same anesthesia and tumor tissue sampling. Tissue blocks (1-cm^3^) were then processed for HE staining and routine pathologic observation by light microscopy. The rest of tumor tissue blocks (1-mm^3^) were subjected to the identical procedures for TEM analysis. Other 6-mice in each group were continuously fed for above-mentioned tumor volume inhibition analysis.

### Statistical Analysis

Statistical analyses were performed using SPSS for windows 11.0. Data were presented as mean ± S.D, and were subjected to analysis using one-way ANOVA, followed by multiple comparisons among test groups or by Dunnett's test for comparisons between test and control groups.

## Results

During the whole experiment, SPEF exposure was well tolerated in all mice. No obvious abnormality in behavior or gross anatomy was observed and no animal death occurred in any groups due to anesthetics or SPEF exposure.

### In Vitro Cytotoxicity of SPEF

**Figure 2:**
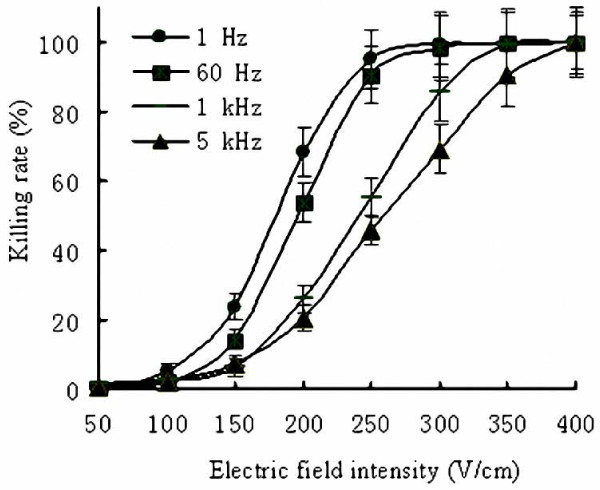
**The cytotoxicity of SPEF with different frequencies and electric field intensity on SKOV3**. Each point on the figure represents the mean value of three independent experiments. For each line, SPEF with a given frequency and appropriate electric field intensity can achieve similar cytotoxicity until reach a plateau of maximum cytotoxicity (approx. 100%).

MTT assay showed that cytotoxicity depended on pulse frequencies and electric field intensity (Figure 2). From the curve, at a given frequency, cytotoxicity of SPEF increased in parallel with electric field intensity. At a given intensity, SPEF with frequency at 1 Hz showed the strongest cytotoxicity among four groups; increased frequency led to decreased cytotoxicity, presented as the curve of cytotoxicity shifted to the right. We could find that higher repetition frequencies seem to require intensive electric field intensity to obtain the maximum cytotoxicity. SPEF with a given frequency and intensity can achieve similar cytotoxicity until reached a plateau of maximum cytotoxicity (approx. 100%). Typically, when frequency reached to 5 kHz, SPEF with intensive energy could also achieve similar cytotoxicity in comparison to low frequency SPEF with weak intensity.

### In Vivo Antitumor Efficiency of SPEF

**Figure 3:**
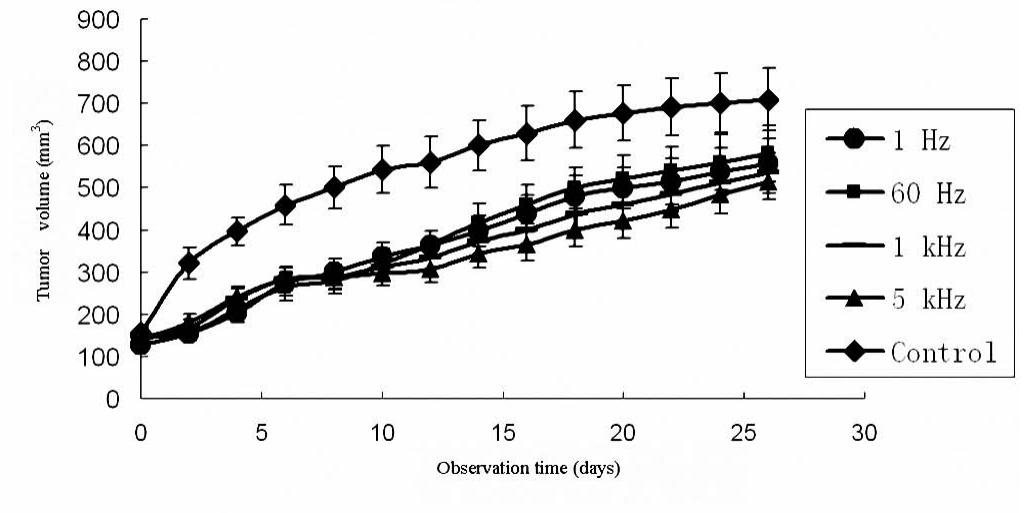
**Tumor volume and growth curve at different observation time among groups**. Each point on the figure represents the mean ± S.D. of six mice. SPEF with different frequencies showed significant antitumor efficiency in comparison to the control group (Dunnett's test, all P < 0.05). However, there was no difference in tumor responses among test groups (one-way ANOVA, all P > 0.05).

Tumor volume and growth curve at different observation time were recorded and compared among test and control groups (Figure 3). Each point on the figure represented the mean value of six mice. At he time of the 26^th ^day, tumor volume of test groups and volume inhibition rate were 557.5 ± 59 mm^3 ^and 26.2% (corresponding to SPEF with frequency of 1 Hz), 581.2 ± 67 mm^3 ^and 23% (60 Hz), 534.5 ± 48 mm^3 ^and 29.2% (1 kHz), 513.9 ± 42 mm^3 ^and 31.9% (5 kHz), while tumor volume in control group was 701.3 ± 74.2 mm^3^. Tumor growth in test groups were significantly lower than that in control group (Dunnett's test, all P < 0.05), indicated that SKOV3 implanted tumor was inhibited evidently by SPEF with different frequencies. On the contrary, multiple comparisons showed no significant difference among test groups (one-way ANOVA, all P > 0.05), indicated that SPEF with different frequencies had similar antitumor efficiency.

### Routine Pathologic Observation

**Figure 4:**
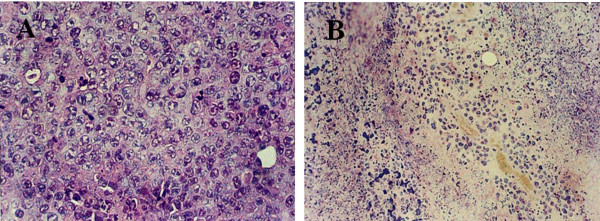
**Routine pathologic observation of SKOV3 subcutaneous implanted tumor in BALB/c nude mice**. 4A. Cancer tissue in the control group grew actively and presented with sheet distribution, high cellularity of cancer cells, multinucleate cancer cells and increased signs of pathologic mitosis. (HE × 400). 4B. Three days after exposure to SPEF (5 kHz), extensive necrosis could be seen in cancer tissue. (HE × 200).

Cancer tissue in the control group grew actively and presented with sheet distribution, high cellularity of cancer cells, multinucleate cancer cells and increased signs of pathologic mitosis (Figure 4A). Three days after exposure to SPEF (5 kHz), extensive necrosis could be seen in cancer tissue (Figure 4B).

### Ultrastructural Observation

The following ultrastructural changes manifested the irreversible damage of tumor cells in response to SPEF exposure.

**Figure 5:**
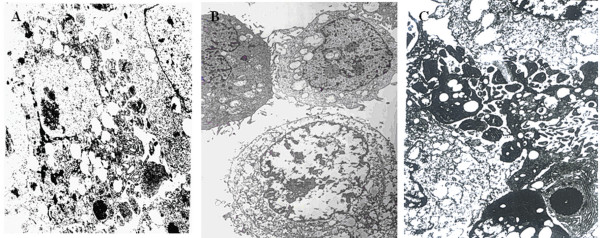
**Microphotos of SKOV3 cells under TEM observation**. 5A: Abundant mitochondria and nucleoli with increased karyoplasm ratio in non-exposure cells. (TEM × 3500). 5B: In response to SPEF exposure (1 kHz), SKOV3 plasma membrane and karyotheca was disintegrated, subcellular organelles such as mitochondria, endoplasmic reticulum and nucleus were cavitated and swollen. (TEM × 3500). 5C: Typical characteristic of apoptosis was further induced by SPEF exposure (5 kHz). (TEM × 10000).

**Figure 6:**
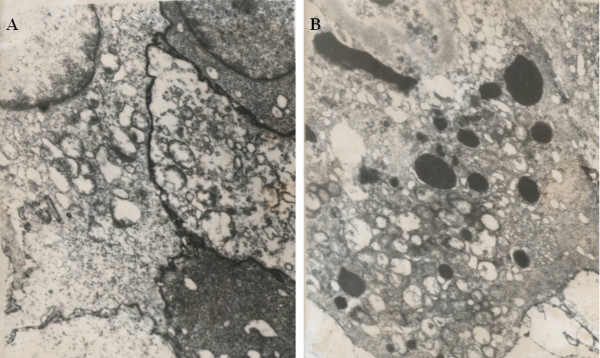
**Microphotos of SKOV3 subcutaneous implanted tumor under TEM observation. (TEM × 10000)**. 6A: In response to SPEF exposure (1 kHz), the integrality of cell membrane was destroyed along with pyknosis, karyorrhexis and karyolysis. 6B: Typical characteristic of apoptosis was further induced by SPEF exposure (5 kHz).

TEM observation showed abundant mitochondria and nucleoli with increased karyoplasm ratio in non-exposure SKOV3 (Figure 5A). However, in response to SPEF exposure (1 kHz), SKOV3 plasma membrane and karyotheca was disintegrated, subcellular organelles such as mitochondria, endoplasmic reticulum and nucleus were cavitated and swollen (Figure 5B). Similarly, the integrality of cell membrane also was destroyed along with pyknosis, karyorrhexis and karyolysis in SKOV3 implanted tumor (1 kHz) (Figure 6A). In addition to so much irreversible damage, typical characteristic of apoptosis was further induced by SPEF exposure (5 kHz) (Figure 5C and 6B).

## Discussion

### Pulsed Electric Fields in Tumor Electrical Treatment

Recent advance in biomedical engineering has enabled great progress in pulsed electric fields. Microsecond electric pulse with weak intensity can create reversible membrane electroporation to enhance drug-uptake such as chemotherapeutic drugs, antibody and exogenous macromolecule substance which are impermeable under normal conditions. Reversible electroporation can be used in electrochemotherapy to sensitize cancer cells to anticancer drugs or in transcutaneous drug delivery [3]. An European project (European Standard Operating Procedures of Electrochemotherapy, ESOPE) had proven electrochemotherapy to be an easy, highly effective, safe and cost-effective approach for the treatment of cutaneous and subcutaneous tumors of different malignancies [21,22]. Furthermore, Microsecond electric pulses with intensive energy often induce irreversible membrane electroporation which can be used to implement tumor ablation directly without any drugs [5]. On the other hand, when shorten the duration of the pulse from microsecond to nanosecond, nanosecond electric pulse can penetrate the intact plasma membrane to impose electric force on multiple subcellular structures and induce multiple biophysical effects known as intracellular electromanipulation, which can be used in cancer treatment, gene therapy and wound healing [7].

The application of microsecond or nanosecond electric pulse in caner treatment has been the focus and was widely accepted by researchers. However, to our knowledge, few researchers have investigated the biophysical effects regarding the combined application of microsecond and nanosecond duration electric pulse in cancer treatment. Recently, according to an "online release" appeared on the official website of the Frank Reidy Research Center for Bioelectrics in Old Dominion University, a dual pulsing system combining long pulses, which open pores in the outer cell membrane, and short pulses, that affect intracellular structures and molecular transport, to enhance gene delivery to the nucleus, was under development [23].

For the first time, we reported the use of both types of electric pulse in this study. We were convinced that the application of this new technology would be of great value in clinical medicine. SPEF was a kind of electric energy transmission method which was unique from existing micro- or nano-second electric pulse. It was designed to combine micro- and nano-second electric pulse into one integral exponential decayed pulses simultaneously. SPEF had a fast rise-time at nanosecond level, containing a large spectrum of high electromagnetic frequencies, and a long queue at microsecond level with low electromagnetic frequencies. According to Hofmann's electric circuit cell model [24], microsecond pulse with low frequency electromagnetic fields mainly cause energy deposition in cell membrane and create reversible or irreversible electroporation, nanosecond pulse with high frequency electromagnetic fields can scramble subcellular organelles [1,7]. Therefore, the two components of SPEF allow combined killing effects on cell membrane and on the subcelluar organelles simultaneously. We had confirmed that SPEF with different parameters could exert different biophysical effects [8-13] and destroy target area in an intensity-dependent manner [14].

Patients received electrochemotherapy often associated with unpleasant sensations, mainly result from low-frequency (1 Hz) electric pulse induced muscle contractions [25]. Zupanic et al., demonstrated that pulse repetition frequency had a close relation to muscle contraction, increasing the pulse repetition frequencies which higher than tetanic could reduce unpleasant sensations that occur in electrochemotherapy [15]. With respect to electroporation efficiency, Pucihar et al., discovered the absence of a direct influence of high frequency microsecond electric pulse on the uptake into electropermeabilized cells in vitro [16]. Furthermore, similar results by Miklavcic et al., also described that high frequency microsecond electric pulse actually didn't decreased its antitumor efficiency in electrochemotherapy [17]. On the other hand, pain sensation during electrochemotherapy also involves pulse parameters such as pulse amplitude, number, duration, and shape of the pulses [18]. Generally, for efficient electrochemotherapy, electric pulses of appropriate parameter must be delivered to the target tissues. However, due to the specificity of SPEF, little data were known regarding the effect of different pulse frequencies on in vitro and in vivo antitumor efficiency by the dual component type of pulse in SPEF.

### In Vitro and In Vivo Antitumor Efficiency of SPEF

In this paper, we studied in vitro and in vivo antitumor efficiency by SPEF with different frequencies and electric field intensity. In vitro test showed that cytotoxicity of SPEF increased in parallel with electric field intensity. SPEF with a given frequency and electric field intensity could achieve similar cytotoxicity until reached a plateau of maximum cytotoxicity (~100%). Increased pulse repetition frequencies didn't significantly reduce the maximum value of the cytotoxicity even at the highest frequency (5 kHz). However, higher electric field intensity seemed to be required to obtain the maximum cytotoxicity with the increased repetition frequency of electric pulses. SPEF with 1 Hz or 5 kHz could achieve similar cytotoxicity when accompanied by appropriate electric intensity. Previous research performed by Pucihar et al., also revealed similar result, DC3F cell suspension was exposure to microsecond duration electric pulse, they proved that even if the frequencies reached to 8.3 kHz, electroporation efficiency and the uptake into in vitro electropermeabilized DC3F cells remained unchanged [17].

In the following test, SPEF with increased frequencies (1, 60, 1 000, 5 000 Hz) and fixed electric field intensity (250 V/cm) were introduced into SKOV3 subcutaneous implanted tumor in BALB/c nude mice. In vivo antitumor assay showed that SPEF with different frequencies had significant antitumor effect in comparison to the control group. However, we did not observe any difference in antitumor efficiency among different frequencies even if the frequencies reach 5 kHz. Daskalov et al., also revealed similar result, electrochemotherapy with high frequency pulses was performed on basal cell and spin cell carcinoma and on melanoma metastases in patients. No difference in tumor responses was observed between 1-Hz and 1 kHz bipolar rectangular pulses [26]. Heller et al., also reported that the benefits from the use of high frequency electric pulses including overcoming the resistance of target tissue and reaching effective depth of interaction [27]. Furthermore, Chang and coworkers had also reported high efficiency gene transfection by membrane electroporation using a radio-frequency electric field (40-kHz frequency) [28].

Further study confirmed that SPEF with 5 kHz could induce apoptosis observed by TEM both in vitro and in vivo. We proposed that induced apoptotic effect was probably a consequence of scramble effects on the target subcellular organelles by the nanosecond pulse component in high frequency SPEF. Our previous study also demonstrated that SPEF with appropriate parameters could trigger cell apoptosis through intracellular calcium electromanipulation [13]. Another study by Weaver et al., also revealed that high frequency electromagnetic fields could cause mitochondrial electropermeabilization, inhibit energy generation and cell proliferation, further induced apoptosis [1].

### Potential Use of High Frequency SPEF in Electrochemotherapy

Motor nerves of skeletal muscle in most mammals were mainly composed of myelinated nerve fibres. The data on the maximum frequency of generated action potentials were calculated to be about 400~2500 Hz (inverse value of the duration of the action potential and the refractory period) regarding to the absolute refractory period which depending on the axonal diameter, myelinated thickness and the number of myelinated nerve fiber [29]. As we know, electrical stimulation during absolute refractory period lead to null muscle contractility. Practically, electric pulse with a train of 8-pulses at standard repetition frequency of 1 Hz has been typically used in traditional electrochemotherapy for many years [17]. However, it deserves to be specially noted that, the limitation of such stimulus is that each individual pulse delivered consecutively can become an active stimulus, activate motor nerves in neuromuscular junctions around the electrodes and then generate an isolated muscle contraction. As reported in the literature, approximately 40 Hz electric stimulation will fuse successive muscle contractions into smooth motion-tetanic contraction [29]. With pulse repetition frequencies below the frequency of tetanic contraction (approx. 40 Hz for humans), increasing the frequency of electric pulses would shorten the delay between two consecutive muscle contractions and subsequently increased muscle contraction. Ultimately provoke sustained contraction of muscle (tetany) and painful burning sensation in electrochemotherapy [15]. In addition, low frequency electric pulse can directly irritate nerve endings of pain receptors to cause intensive pain. Therefore, researchers now advocate discarding the use of low frequency electric pulse for electrochemotherapy [17]. Interestingly, however, the benefits of this unique characteristic of low frequency electric pulse had been widely used in neuromuscular electrical stimulation for patients suffered from peripheral facial paralysis [30].

The aim of our study was to employ high frequency electric pulse for tumor electrical treatment. We speculated that when the delay between two consecutive electric pulses was shorter than the duration of the action potential and the refractory period, also can be interpreted as, the pulses repetition frequencies were higher than the frequency of tetanic contraction (approx. 40 Hz). In this case, single or multiple electrical pulses in one repetition frequency will skip out of the absolute refractory period which is essential to generate action potentials and initiate muscle contractility. Subsequently, achieve the purpose of reducing sustained contraction of muscle (tetany) and relieve painful sensation. Miklavcic et al., also reported that at pulse frequencies higher than 2000 Hz, the muscle torque was similar to that after application of a 1 Hz pulse train (a typical electrochemotherapy protocol) [17]. It is thus evident that, increasing the repetition frequency even far exceeds the frequency of tetanic contraction, electric pulse doesn't sharpen the pain in tumor electrical treatment.

It should be highlighted that Marty and colleagues newly developed a machine called Cliniporator™ (Igea s.r.l. Carpy, Italy) that had been certified to use on patients in the European market along with the ESOPE project for the treatment of cutaneous and subcutaneous tumors of different malignancies. It can generate the 5 kHz microsecond electric pulses which is now being used prevalently in the most of electrochemotherapy treatments [21,22]. More recent studies by Marty et al., [21] and Mir et al., [22] and Sersa et al., [31] showed in their clinical studies, that electrochemotherapy with Cliniporator™ at a repetition frequency of 5 kHz could reduce the number of contractions to one and there was no difference in the level of pain when compared to 1 Hz. Furthermore, they found that the 5 kHz repetition frequency of the applied electric pulses resulted in statistically significantly better antitumor effect than the 1 Hz repetition frequency. Their studies also suggested that the use of 5 kHz frequency had the advantages in shortening the treatment time, especially in the treatment of multiple tumor nodules.

## Conclusion

We demonstrated that, SPEF with high repetition frequency could also achieve similar levels of in vitro and in vivo antitumor efficiency which could be used to reduce unpleasant sensations that occurred in tumor electrical treatment. In addition, rich components of nanosecond pulse contained in SPEF with high frequency electromagnetic fields (5 kHz) could induce cell apoptosis and provided complementary antitumor effect for the marginal regions with weak electric fields. Our research proposed potential applications and feasibility of using high frequency SPEF in clinical cancer treatment.

Nevertheless, it should be noted that this study examined only in vitro and in vivo antitumor effect of SPEF with various frequencies. However, effects of pulse repetition frequencies on biomechanical properties of skeletal muscle and on pain perception threshold remained to be further clarified. In future study, in order to integrate current antitumor investigation with biomechanical experiment and extend its perspective clinical applications, we should take this limitation into consideration and try to perform in vivo biomechanical test and pain threshold measurement in response to SPEF with different frequencies.

## Abbreviations

SPEF: Steep Pulsed Electric Fields; SPF: Specific Pathogen Free; TEM: Transmission Electron Microscopy.

## Competing interests

The authors declare that they have no competing interests.

## Authors' contributions

YXJ supervised the project, conceived the study, provided financial assistance for the study, carried out cell culture experiments, and data analysis. LJ elaborated the design and performed tumor formation in BALB/c nude mice, determined frequency related antitumor efficiency, and TEM observation. SCX engineered the hardware to perform SPEF stimulation throughout the experiment. ZFY helped to revise the manuscript. HLN co-funded and participated in its design, coordination. All the authors had given final approval for publication. YXJ and LJ were considered first authors since both authors contributed equally to this work.
